# Role of Nociceptin/Orphanin FQ-NOP Receptor System in the Regulation of Stress-Related Disorders

**DOI:** 10.3390/ijms222312956

**Published:** 2021-11-30

**Authors:** Massimo Ubaldi, Nazzareno Cannella, Anna Maria Borruto, Michele Petrella, Maria Vittoria Micioni Di Bonaventura, Laura Soverchia, Serena Stopponi, Friedbert Weiss, Carlo Cifani, Roberto Ciccocioppo

**Affiliations:** 1 School of Pharmacy, University of Camerino, Via Madonna Delle Carceri 9, 62032 Camerino, Italy; massimo.ubaldi@unicam.it (M.U.); nazzareno.cannella@unicam.it (N.C.); annamaria.borruto@unicam.it (A.M.B.); michele.petrella@unicam.it (M.P.); mariavittoria.micioni@unicam.it (M.V.M.D.B.); laura.soverchia@unicam.it (L.S.); serena.stopponi@unicam.it (S.S.); carlo.cifani@unicam.it (C.C.); 2 Department of Molecular and Cellular Neuroscience, The Scripps Research Institute, La Jolla, CA 92037, USA; bweiss@scripps.edu

**Keywords:** nociceptin/orphanin FQ, NOP receptor, stress, addiction

## Abstract

Nociceptin/orphanin FQ (N/OFQ) is a 17-residue neuropeptide that binds the nociceptin opioid-like receptor (NOP). N/OFQ exhibits nucleotidic and aminoacidics sequence homology with the precursors of other opioid neuropeptides but it does not activate either MOP, KOP or DOP receptors. Furthermore, opioid neuropeptides do not activate the NOP receptor. Generally, activation of N/OFQ system exerts anti-opioids effects, for instance toward opioid-induced reward and analgesia. The NOP receptor is widely expressed throughout the brain, whereas N/OFQ localization is confined to brain nuclei that are involved in stress response such as amygdala, BNST and hypothalamus. Decades of studies have delineated the biological role of this system demonstrating its involvement in significant physiological processes such as pain, learning and memory, anxiety, depression, feeding, drug and alcohol dependence. This review discusses the role of this peptidergic system in the modulation of stress and stress-associated psychiatric disorders in particular drug addiction, mood, anxiety and food-related associated-disorders. Emerging preclinical evidence suggests that both NOP agonists and antagonists may represent a effective therapeutic approaches for substances use disorder. Moreover, the current literature suggests that NOP antagonists can be useful to treat depression and feeding-related diseases, such as obesity and binge eating behavior, whereas the activation of NOP receptor by agonists could be a promising tool for anxiety.

## 1. Introduction

Nociceptin/orphanin FQ (N/OFQ) is a neuropeptide composed of 17 residues with the sequence FGGFTGARKSARKLANQ which activates the nociceptin opioid-like receptor (NOP). NOP receptor is a seven transmembrane-spanning G protein-coupled receptor (GPCR) whose ligand was unknown until 1995, when it was finally deorphanized by two different research teams at the same time [1,2]. NOP was identified about 2 years earlier as a result of a homology analysis showing that the NOP amino acids sequence was about 50% identical to MOP, DOP and KOP opioid receptors [3]. This homology is also functional since, like the other opioid receptors, NOP couples to Gi- and Go-coupled receptors and its activation results in adenylyl cyclase inhibition, activation of MAP kinase, enhancement of potassium conductance and closing of voltage-sensitive calcium channels [3,4]. Similarly, the N/OFQ shares sequence homology with the other opioid peptides. Indeed, N/OFQ, originates from a larger precursor molecule called prepronociceptin (PPNOC). The PPNOC gene is organized in four exons intermixed by three introns. This gene originates a transcript of 1300 bp that encodes a single copy of nociceptin. This precursor shares sequence homology with the precursors of other opioid neuropeptides such as prodynorphin and preproenkephalin [5]. However, despite these structural and genetic similarities, N/OFQ does not activate MOP, KOP or DOP receptors and opioid neuropeptides do not activate the NOP receptor. On the contrary, in rodent models, the N/OFQ system elicits an antagonistic action on two of the most important opioid actions: reward and analgesia. It was shown that NOP activation by N/OFQ reverses opioid-mediated analgesia [6,7] and antagonizes the rewarding effect of opiates and other drugs of abuse as demonstrated in place conditioning and self-administration experiments [8,9,10]. 

Pain was the first area of research in which the physiological role of N/OFQ-NOP receptor system was studied. In fact, the term nociceptin derived from the finding of pronociceptive behaviors following intracerebroventricular (i.c.v.) microinjection of the peptide [1]. Studies performed afterward showed that this effect was due to the ability of N/OFQ to antagonize the stress-induced analgesia caused by the surgical procedure used in the experiment [11]. Evidence accumulated so far indicates that, in rodents, N/OFQ exerts a bimodal modulation of pain acting through central and peripheral mechanisms. It induces anti-opioid hyperalgesic effects when acting within the brain, while it produces analgesic effects in the spinal pain pathways [12,13,14,15,16]. In primates the role of the N/OFQ-NOP receptor system appears to be different and its activation mediates analgesic effect both at spinal and brain levels.

The NOP receptor is widely expressed in the brain, including regions such as the hypothalamus, cingulate and piriform cortices, periaqueductal gray (PAG), amygdala, bed nucleus of the stria terminalis (BNST), locus ceruleus (LC), endopiriform nucleus, raphe nuclei, hippocampus, substantia nigra and ventral tegmental area (VTA) [3,17]. The localization of N/OFQ assessed by immunohistochemistry overlaps substantially with the expression of PPNOC mRNA. N/OFQ localization is restricted to BNST, amygdala, thalamus, some cortical regions, striatum, substantia nigra, VTA and hypothalamus [18]. The more limited distribution of the peptide compared to the receptor suggests that the place of synthesis of N/OFQ partly differs from its site of action.

Consistent with its anatomical localization in the brain, the N/OFQ-NOP receptor system is involved in different physiological and motivational processes including learning and memory, pain, anxiety, stress, depression, addiction and ingestive behaviors. For instance, administration of N/OFQ in the dorsal region of the hippocampus impairs spatial learning, although this effect was detected at very high doses of the peptide [19], whereas, i.c.v. injection of the peptide elicits an anxiolytic effect across many different experimental paradigms in rodents [13,20] and it induces acute hyperphagia [21,22,23]. In the periphery NOP is localized in the spleen, liver and gastrointestinal tract [24], while N/OFQ is also present in the kidney and ovaries. N/OFQ produces vasodilation, negative chronotropic and inotropic effects, inhibition of gastrointestinal motility, sepsis, inflammation, and antitussive effects [25,26,27,28,29].

Over the years a considerable number of pharmacological agents that activate or block NOP have been developed allowing the characterization of the biological role of the N/OFQ system and to evaluating its potential as therapeutic target in various diseases. Nonpeptide NOP receptor agonists and antagonists able to cross the brain-blood barrier (BBB) have shown a good safety and tolerability profile although their clinical development was hindered by the identification of important side effects. The first NOP agonist to enter clinical trials was SCH-486757 that was investigated as an antitussive agent [30]. SCH-486757 showed a greater efficacy compared to the opioid codeine in treating cough [31]. Unfortunately, it also produced a significant sedation and for this reason its development as antitussive was discontinued. Another example is represented by Ro 64-6198 a NOP agonist extensively studied in preclinical models. Ro 64-6198 showed a significant anxiolytic profile across several paradigms and rodent species [32]. This compound was also used to investigate the modulation of pain sensitivity by the nociceptin system [33,34] and its role in drug abuse [35]. However, despite a good BBB penetrance [36], the clinical development of Ro 64-6198 was hindered by a poor bioavailability [37]. 

NOP antagonists have attracted interest as potential therapeutic targets for the treatment of psychiatric and neurological disorders such as depression, Parkinson’s disease, obesity, schizophrenia-associated cognitive impairment, drug abuse and neuropathic pain. For instance, LY2940094 (aka BTRX-246040), a selective and potent NOP antagonist recently developed by Eli Lilly [38], completed phase II clinical trials for the treatment of the major depressive disorder (MDD) and alcohol addiction, Other two NOP receptor antagonists, MK-5757 and JTC-801, entered clinical trials to treat cognitive impairment symptoms associated to schizophrenia and neuropathic pain, respectively (for review see [39]). Selective radioligands like ^11^C-NOP-1A and ^18^F-MK-0911 for Positron Emission Tomography (PET) imaging have been also developed [40,41]. These radioligands, suitable for clinical studies will allow the quantification of NOP in the brains of psychiatric patients and helping to characterize the role of NOP in mental disorders.

As mentioned above, activation of NOP attenuates the rewarding effect of opiates and other drugs of abuse (for review see [42]). Hence, drugs that simultaneously activate NOP in addition to the canonical opioid receptors (especially MOP), have the potential to act as an analgesic with moderate abuse liability. Based on this hypothesis, research programs aimed at characterizing the pharmacological properties of mixed MOP/NOP agonists have attracted attention [43,44]. Two drugs with this mixed pharmacological profile are buprenorphine and cebranopadol. Buprenorphine is a MOP/NOP partial agonist and a KOP/DOP antagonist [45,46]. It is approved for the treatment of chronic pain and heroin addiction and is emerging as a promising drug to treat alcohol and cocaine dependence [47,48,49,50,51,52]. As a result of this research effort it was recently demonstrated that buprenorphine reduced cocaine consumption by the co-activation of NOP and MOP receptors [47]. Cebranopadol is an opioid analgesic, full agonist at MOP, DOP and NOP receptors and a KOP partial agonist, which preferentially binds NOP and MOP [53,54]. Cebranopadol showed anti-addictive properties and reduce cocaine self-administration in the rat [55]. As with buprenorphine, the cebranopadol effect was prevented by concomitant blockade of MOP and NOP receptors [55]. Altogether, these data demonstrate that bifunctional MOP/NOP agonists have analgesic effects but lower abuse potential compared to classical MOP selective ligands. Moreover, they show therapeutic potential in the treatment of drug abuse [43].

In addition to pain and addiction the N/OFQ-NOP receptor system is tightly linked to the modulation of mood state and stress response [32,56]. In the present review we provide an updated view of the role of this peptidergic system in the regulation of stress-related disorders and psychiatric conditions such as anxiety, depression, substance use disorders and maladaptive feeding behaviors. The therapeutic potential of NOP agonists and antagonists is systematically presented.

## 2. Neurobiology of the N/OFQ-NOP Receptor System

The binding of N/OFQ on the NOP receptor produces the activation of a classical Gi/o signaling, leading to the dissociation of the G protein in a Gα and a Gβγ subunit. The Gα subunit mainly acts by inhibiting adenylate cyclase activity and thus reducing cyclic adenosine monophosphate (cAMP) intracellular concentrations and protein kinase A activity. The Gβγ subunit directly activates G protein-coupled mediated inwardly rectifying potassium (Kir3) and inhibits presynaptic calcium channels (Cav2.1, Cav2.2 and Cav2.3). Furthermore, the Gβγ subunit regulates several intracellular signaling kinase cascades, including protein kinase C (PKC), phospholipase A (PLA) and C (PLC), and extracellular signal-regulated kinases 1 and 2 (ERK1/2) [57]. The positive modulation of the postsynaptic Kir3 channel’s function generates neuronal hyperpolarization and firing inhibition, while the blocked of presynaptic Cav2.x calcium channels reduces neurotransmitter release (for an extensive review on the electrophysiological actions of N/OFQ see [58]). Despite sharing the same cellular mechanism of action of classical opioid receptors, NOP differs in its anatomical distribution, which makes its functional role unique. As revealed by several in vitro and in vivo studies, in virtually all brain regions scrutinized these effects are believed to produce a generalized inhibition of neurotransmitters release, including glutamate, gamma-aminobutyric acid (GABA), central monoamines, and neuropeptides (see the next section). However, it should be remarked that the expression of NOP receptors in different neuronal ensembles within the same brain regions could lead to the disinhibition of their outputs in response to N/OFQ. Furthermore, given the adaptability of the N/OFQ-NOP receptor system in response to behaviorally relevant experiences, the net effects of its activation/inactivation could also be regulated by state-dependent physiological and pathological factors such as anxiety, mood disorders and drug addiction (for review see [57,59]. Consistently, neuroanatomical data demonstrated a high expression of N/OFQ and NOP receptor in brain regions of the mesocorticolimbic system, the extended amygdala and the hypothalamus, that are known to subserve the regulation of motivated behaviors, stress response and affection [17,18,60,61,62]. 

### 2.1. N/OFQ-NOP and the Mesocorticolimbic Reward System

**Figure 1:**
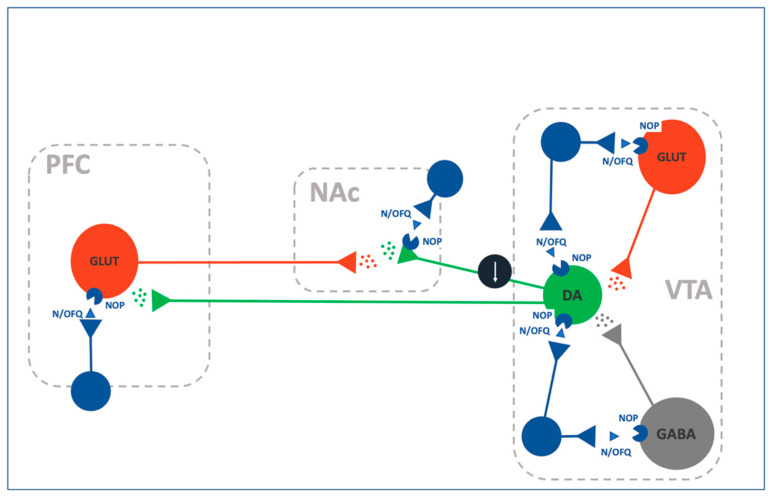
Schematic representantion of the relationship of the N/OFQ-NOP system with other neurocircuitries within the mesocorticolimbic reward system. Prefrontal Cortex (PFC); Nucleus Accumbens (NAc); Ventral Tegmental Area (VTA).

The mesolimbic dopamine (DA, Figure 1) system is a major player in the modulation of reward and reinforcement mechanisms [63,64]. A key step in reward processing consists of the activation of DAergic neurons in the VTA and the subsequent release of this neurotransmitter in the nucleus accumbens (NAc) [63,64]. 

One of the first study implicating the N/OFQ-NOP receptor system in the modulation of mesolimbic activity was published few months after the deorphanization of the system by Murphy et al. (1996). In this study, using in vivo microdialysis in rodents, the authors demonstrated that i.c.v. administration of N/OFQ decreases DA extracellular concentrations in the NAc [65]. These initial results were mirrored by experiments in which N/OFQ was directly applied into the VTA and in which was shown that a direct modulation of DAergic cells in this region is responsible for reduced DA release in the NAc [10]. Accordingly, slice electrophysiological recordings demonstrated a direct (TTX-insensitive) inhibition of VTA DAergic neuronal activity induced by N/OFQ perfusion. These effects are mediated by NOP receptors and appear to depend on enhancement of potassium conductance [66,67]. This conventional mechanism of action has been recently questioned in a study proposing the possibility that N/OFQ may attenuated DA transmission through indirect mechanisms involving presynaptic GABA transmission [68]. However, more conclusive experiments are needed to support this alternative hypothesis. Thus, at present there is little debate that effects produced by N/OFQ in VTA DAergic neurons are primarily mediated by a direct activation of NOP receptors located in these cells. Accordingly, a recent publication demonstrated that NOP receptors expressed in VTA DAergic neurons are necessary and sufficient for mediating the reduced motivation for sucrose produced by treatment with the NOP agonist SCH221510, suggesting that the decreased DA tone produced by N/OFQ mainly depends on NOP receptor expressed on VTA DAergic cells [62]. 

NOP receptors are also expressed in DAergic presynaptic terminals and postsynaptically within the NAc, further increasing the complexity of the N/OFQ modulations of mesolimbic DA functions [69]. At this level, N/OFQ treatment produced a decrease in tyrosine hydroxylase (TH) phosphorylation, DA synthesis and postsynaptic DA receptor D1 signaling [69]. On the other hand, in vivo microdialysis and ex vivo fast scan cyclic voltammetry studies revealed that N/OFQ application within the NAc has little or no basal effects on DA accumulation and release [68,70]. Altogether these data point to a predominant role of the VTA in mediating the effects of N/OFQ in DA release. Nevertheless, NOP activation within the NAc counteracted the increase in DA extracellular concentration produced by an acute cocaine exposure, suggesting that N/OFQ could exert a state-dependent modulation of DA release depending on the system activation rate [70]. 

The generalized inhibitory activity of N/OFQ on DA release prompted to intuition that receptor blockade would instead increase its extracellular levels. Guided by this hypothesis few studies have investigated the effect of NOP blockade on DA transmission. 

The first evidence showed that selective NOP antagonism prevents the inhibition of DA elicited evoked by intracranial administration of N/OFQ [71,72,73]. Noteworthy, UFP-101, a peptidic NOP antagonist, in addition to blocking the effect of N/OFQ on DA release, induced per se a mild but lasting suppression of mesolimbic DA [73]. In contrast subsequent studies found that peripheral administration of J-113397, another selective NOP antagonist, also enhanced DA release in the NAc and increased VTA DAergic neuronal activity [62,74]. However, these results should be carefully evaluated since the effects of J-113397 in releasing DA were present also in NOP receptor knock-out (NOP−/−) mice, suggesting that this effect is, at least in part, independent from the NOP receptor [74]. 

Finally, in a series of interesting studies it has been reported that blockade of NOP receptors reduced glutamate and enhanced GABA release in the nigrostriatal pathway [75,76,77,78]. Based on these data it is tempting to hypothesize that inhibition of mesolimbic DA transmission by NOP agonists is dependent upon direct hyperpolarization of VTA DAergic neurons mediated by NOP receptors expressed on these cells. While reduction of DA transmission by NOP antagonist depends on the removal of N/OFQ inhibition on GABA release, subsequent enhancement of GABA transmission resulting in the hyperpolarization of VTA DA neurons. This heuristic hypothesis implies that under physiological condition there is a tonic inhibition of VTA GABAergic neurons by N/OFQ. This tonic inhibition is removed by administration of the NOP antagonists. Whereas, in case of exogenous administration of agonists VTA transmission is inhibited by the activation of NOP receptors directly expressed by DAergic neurons. This view is corroborated by recent data showing that modulation of reward-seeking by VTA neurons is controlled by state-dependent on activation of the N/OFQ system [62,74]. 

### 2.2. N/OFQ and the Extrahypothalamic Stress System

Corticotropin-releasing factor (CRF) is the primary mediator of stress in mammals. When released it activates the HPA axis via stimulation of CRF1R in the anterior pituitary, evoking adrenocorticotropic hormone (ACTH) secretion and subsequent glucocorticoid production and release from the adrenal gland [79,80]. However, CRF acts also through extrahypothalamic circuitries mediating negative affective state and mood disorders associated with stress and drug abuse [81,82]. Consistent, with the multiple roles of this system, CRF and CRF1 receptors have been identified in several brain regions. Indeed in addition to the paraventricular nucleus of the hypothalamus (PVN), a high concentration of CRF-containing cell bodies has been identified in the neocortex, thalamus and the VTA [83,84]. CRF and CRF1R are also expressed in the bed nucleus of the stria terminalis (BNST) and in the central nucleus of the amygdala (CeA) that together with the shell of NAc establish the extended amygdala [85], a crucial component of the extrahypothalamic brain stress system [86,87,88]. Few studies demonstrated that administration of N/OFQ mediates pro-stress response by activating the hypothalamic-pituitary-adrenal (HPA) axis (causing an increase of adrenocorticotropin hormone and corticosterone levels in the plasma) [89]. On the other hand, N/OFQ exerts functional antagonism towards the extrahypothalamic CRF and CRF1R, exerting antistress and anxiolytic actions [81,82,90,91]. 

**Figure 2:**
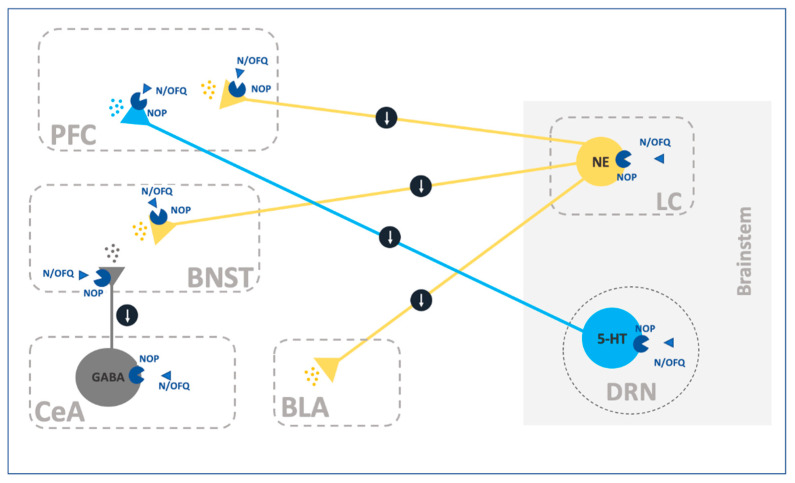
Schematic representantion of the relationship of the N/OFQ-NOP system with other neurocircuitries within the extrahypothalamic stress system. Prefrontal Cortex (PFC); Bed Nucleus of the Stria Terminalis (BNST); Central Nucleus of Amygdala (CeA); BasoLateral Nucleus of Amygdala (BLA); Locus Coeruleus (LC); Dorsal Raphe Nucleus (DRN).

Given the anxiolytic and anti-stress role of N/OFQ much attention has been focused on its function in the modulation of extrahypothalamic networks (Figure 2). Studies demonstrated that N/OFQ and NOP receptors are strongly expressed in the CeA [17,18], and microinjections of the peptide into this nucleus decreased anxiety both in naïve and stressed rats [92,93]. Several studies analyzing the electrophysiological mechanism by which N/OFQ modulates the activity of the CeA have been published in the last two decades. In 2006, Roberto and Siggins demonstrated that N/OFQ perfusion decreased GABA transmission in CeA neurons through a presynaptic mechanism of action [94]. Similarly, N/OFQ treatment has also been linked to a reduction in the glutamatergic inputs to CeA cells [95].These effects were accompanied by a direct postsynaptic hyperpolarization in a subset of the CeA neurons, consistent with the activation of an inwardly rectifying potassium conductance [94,96,97,98]. Intriguingly, when the recordings were selectively aimed at the periaqueductal gray (PAG)-projecting centromedial amygdala (CeM) neurons the percentage of neurons inhibited by N/OFQ dramatically increased [98], supporting the assumption that NOP agonists could exert their anxiolytic effects by blunting the amygdala outputs. The N/OFQ-NOP receptor system is prone to adapt following behavioral relevant experiences, such as stress or consumption of substances of abuse. For instance, acute exposure to social defeat stress enhanced the expression of NOP receptors but not that of PPNOC mRNA within the amygdala in rats [99]. In partial agreement, rats exposed to acute restraint stress showed an increase in the levels of N/OFQ and NOP mRNAs within the amygdala [93]. This was confirmed by slice electrophysiological experiments that showed a significantly larger N/OFQ-dependent inhibition of CeA GABAergic signaling in restraint rats compared to controls. Coherently with the upregulation of NOP following stress, treatment with the NOP antagonist [Nphe1]-nociceptin(1–13)NH2, potentiated the GABAergic inputs recorded from the CeA of restraint but not control rats [93]. Similarly, it has been demonstrated that alcohol post-dependent rats expressed an increased inhibition of CeA GABAergic, but not glutamatergic inputs following N/OFQ exposure [94,95]. This set of data suggests that stress and addiction, by modifying the strength of different network components could modify the overall behavioral effects produced by exposure to NOP receptor agonists and antagonists. 

N/OFQ does not bind to CRF receptors, however several lines of evidence suggest that it can act as a functional corticotrophin antagonist [100]. For instance, electrophysiological recordings showed that N/OFQ completely abolished the CRF-dependent increase in CeA GABAergic inhibition through a NOP-mediated mechanism of action [93,101]. Another brain site in which the interaction between CRF and N/OFQ may occur is the BNST, where microinfusion of N/OFQ occluded the anxiogenic-like and or anorexigenic effects of CRF and stress [90,91]. Electrophysiology experiments also demonstrated that N/OFQ reduced the neuronal firing in about 50% of the anterior BNST neurons regardless of their dorso/ventral anatomical location and neuronal subtype [102]. A third area possibly mediating the anxiolytic-like effects of NOP agonists is the basolateral complex of the amygdala (BLA). The BLA has a fundamental role in the acquisition and expression of conditioned fear and an hyperactivation of the BLA has been linked to pathological anxiety [103]. Although lacking significant presence of N/OFQ mRNA, the BLA expresses NOP receptor mRNA and is targeted by N/OFQ-positive fibers [17,18,104]. Electrophysiological recordings from the lateral portion of the BLA demonstrated a mixture of pre- and postsynaptic effects mediated by N/OFQ resulting in a decreased glutamate and GABA release accompanied by a direct postsynaptic inhibition of the vast majority of recorded neurons [96,105]. 

Besides the interplay with CRF in the extended amygdala, N/OFQ also modulates the release of catecholamines that are known to play an important role in stress-related processes. For instance, decreased release of Norepinephrine (NE) in response to N/OFQ application has been consistently reported in neocortical brain slices and synaptosomes [106,107,108,109]. Accordingly, slice electrophysiological recordings showed that N/OFQ activates an inwardly rectifying potassium conductance in the vast majority of locus coeruleus (LC) cells representing the major source of NE in the brain [110,111]. These results were confirmed and further extended by in vivo microdialysis studies in awake rats that reported a decreased release of NE in the prefrontal cortex (PFC) and the amygdala after local or intra-LC microinjections of N/OFQ or synthetic NOP agonists [72,112,113]. Several in vitro and in vivo studies revealed that administration of N/OFQ also dampened the release of serotonin (5-hydroxytryptamine, 5-HT) [107,109,114,115,116]. Moreover, electrophysiological recordings from the dorsal raphe nucleus (DRN), one of the major source of 5-HTergic neurons, revealed that N/OFQ activates inwardly rectifying potassium channels and decreases the firing rate of putative 5-HTergic neurons [111,117,118]. Noteworthy, with the inhibitory effects of N/OFQ on 5-HT release and DRN neuronal activity was enhanced by acute swim stress exposure. This effect was not mediated by a change in NOP receptor expression or affinity but it was mimicked by CRF and was inhibited by CRF1R antagonism [118,119]. These results indicate that the inhibitory effects of N/OFQ on 5-HT neurotransmission are heavily regulated by CRF. Decreases of 5-HT activity following local administration of N/OFQ in the NAc was also reported, indicating a functional role of NOP receptors in the modulation of 5-THergic transmission within this nucleus [116]. 

The generalized inhibitory activity of N/OFQ on monoamine release prompted to the intuition that receptor blockade would instead increase their extracellular levels. Guided by this hypothesis few studies have investigated the effect of NOP blockade on DA, 5-HT and NE release in rodents. 

Initial studies showed that selective NOP antagonism prevented the inhibition of catecholaminergic transmission elicited by intracranial administration of N/OFQ [71,72,73]. Moreover, using various NOP blockers it was observed that antagonism at N/OFQ receptor increased several neurotransmitters release. For instance, intraperitoneal (i.p.) treatment with the NOP antagonist J-113397 (previously known as Compound B) produced a significant increase in NE release in the amygdala [113]. Increase in 5-HT release within the DRN was observed following intra-DRN infusion of the NOP antagonist [Nphe1]-nociceptin(1–13)NH2 [116]. This effect was replicated in stressed (but not unstressed) rats after exposure to the NOP antagonist UFP-101 [118]. Finally, in a series of interesting studies it has been reported that blockade of NOP receptors reduced glutamate and enhanced GABA release in the nigrostriatal pathway [75,76,77,78]. Altogether these findings, on the one hand demonstrate that the N/OFQ system plays a tonic inhibitory role on catecholaminergic and GABAergic transmission. While, on the other hand, the possibility exists that receptor blockade may represent a suitable strategy to enhance these transmissions to possibly treat psychiatric conditions, such as depression, anxiety that are tightly associated with dysregulation of mesocorticolimbic catecholaminergic transmission. Noteworthy, in a recent study it was shown that LY2940094, a highly potent and selective NOP antagonist, occluded the ability of alcohol to stimulate DA release in the NAc [120]. Altogether these data suggest that the link between N/OFQ and stress can be partly linked to its CRF functional antagonism but in part it may depend upon its ability to modulate the catecholaminergic transmission.

### 2.3. N/OFQ-NOP and the Hypothalamic Stress System

**Figure 3:**
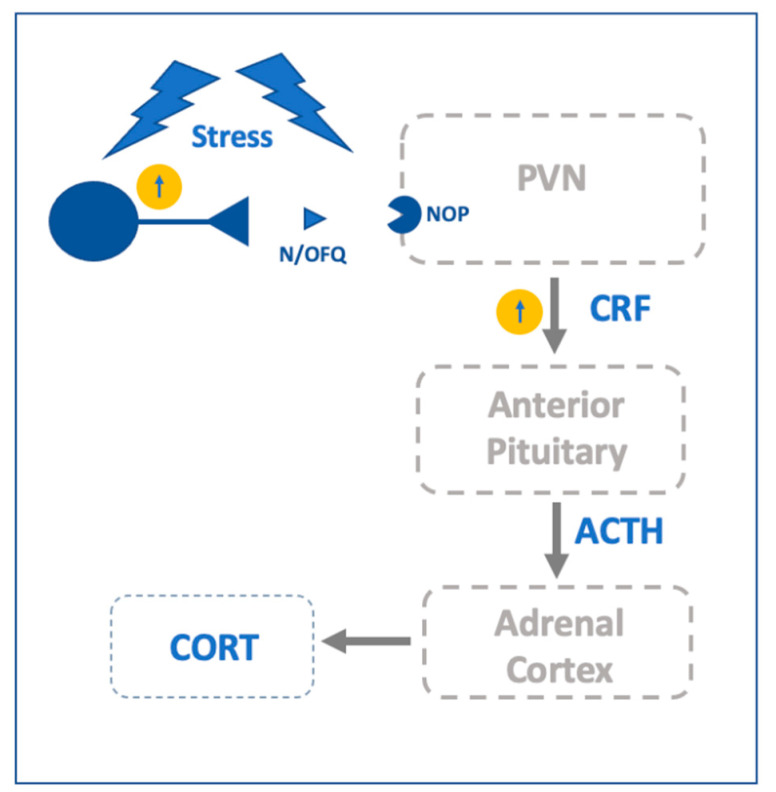
Schematic representantion of the relationship of the N/OFQ-NOP system with other neurocircuitries within the HPA stress axis. Paraventricular Nucleus of the Hypothalamus (PVN).

In apparent contradiction with the anti-stress properties of extrahypothalamic N/OFQ there is substantial literature reporting that N/OFQ stimulates stress-like biological responses in the hypothalamus (Figure 3). N/OFQ and its receptor are highly enriched in several hypothalamic areas, including the PVN [17,18]. The PVN is considered the principal integrator of stress signals and is involved in the modulation of the HPA axis activity [42]. The administration of N/OFQ (i.c.v.) has been linked to an enhanced expression of c-Fos in the PVN [121], promotes the activation of the HPA axis and increases the plasma concentrations of ACTH and corticosterone in naïve and mildly stressed rats [42,89,122,123]. Administration of selective NOP antagonist UFP-101 alone had no significant effect on plasma corticosterone concentration but completely blocked the effect of N/OFQ on corticosterone release. UFP-101 also blocked N/OFQ-induced increase in CRF mRNA and POMC mRNA indicating that N/OFQ-induced HPA axis activation is mediated via central NOP receptors [123]. In further studies using chronic mild stress procedures it was shown that repeated administration of UFP-101 also decrease stress-induced gain in serum corticosterone levels and anhedonia [124]. 

It is important to remark that N/OFQ and NOP receptor expression in the brain are modified by stress exposure [93,99,125], and in rats exposed to more extreme stressful conditions, such as acute restraint stress, N/OFQ administration did not produce any alteration in plasma ACTH and corticosterone concentrations [89]. Intriguingly, i.c.v. administration of the NOP receptor antagonist UFP-101 in the morning was ineffective in unstressed rats, but significantly prolonged the elevations of corticosterone induced by acute restraint stress [123,126]. A functional interplay with the BNST and the hypothalamic stress system has been proposed. Indeed, intra-BNST microinjections of N/OFQ produced augmented plasma concentrations of ACTH and corticosterone. This effect was specific to the BNST, since intra-CeA N/OFQ microinjections were ineffective [127]. Although highly speculative, an intriguing explanation is that N/OFQ could promote stress response by silencing the inhibitory signals that the BNST tonically sends to the PVN [59]. N/OFQ and NOP receptors mRNAs are also enriched in the suprachiasmatic nucleus of the hypothalamus (SCN) [128,129]. The SCN is considered the master regulator of the animal circadian clock, and its modulatory action on the HPA axis is partially mediated by its projections to the PVN [130,131]. Accordingly, plasma levels of stress hormones exhibit circadian fluctuations [132]. Noteworthy, N/OFQ enhanced the potassium conductance and presynaptically reduced glutamatergic and GABAergic inputs toward the vast majority of SCN neurons [128,133]. In brain slice preparations incubation with the highly selective NOP agonist MT-7716 decreased the spontaneous firing rate of SNC neurons [134]. These results have been further confirmed by in vivo experiments that showed a decrease in photic-induced c-Fos activation of the SNC neurons following N/OFQ administration [135]. In addition, treatment with N/OFQ in rats inhibited or stimulated the light-induced phase delay during the night or the day, respectively [135]. Accordingly, in rats, administration of UFP-101 prolonged the increase of corticosterone plasma levels following acute restraint stress exposure in the morning but this effect was absent in the evening [123,126]. 

Altogether, these data stimulated the hypothesis that the activation of NOP could regulate the HPA axis partially through an alteration of animal’s circadian rhythms [82,126]. Additional studies are needed to verify these hypotheses and to better understand the bidirectional interactions occurring between stress and the N/OFQ-NOP receptor system in different pathophysiological situations.

## 3. Role of the N/OFQ-NOP Receptor System in Anxiety and Mood Disorders

The seminal work indicating a role for the N/OFQ-NOP receptor system in the modulation of anxiety was published in 1997, when Jenck and colleagues demonstrated that i.c.v. infusion of non-sedative doses of N/OFQ (0.1–0.3 nmol) induced anxiolytic-like effects in both mice and rats across several behavioral paradigms including the elevated plus maze (EPM), light-dark box test, free exploratory behavior in unfamiliar environment and operant conflict test) [20]. This initial finding was replicated in later studies using central administration of N/OFQ [20,92,136,137,138,139,140,141,142], as well as systemic administration of nonpeptide NOP synthetic agonists [32,37,143,144,145,146,147,148,149,150,151,152]. To the best of our knowledge, only two studies have reported results in contrast with the anxiolytic effect of N/OFQ and NOP agonists. These studies found that i.c.v. (0.01, 0.1, 1.0 nmol), intra-BNST (1.0 nmol) and intra-amygdala (0.1, 1.0 nmol) administration of N/OFQ induced anxiety-like behaviors tested in the open field, elevated plus maze and dark-light neophobic tests [122,127]. It should be considered, however, that N/OFQ causes the impairment of motor skills starting from 0.1 nmol i.c.v. [153]. The motor effect of N/OFQ is biphasic, it is stimulatory at very low concentrations (005–0.05 nmol) but it is inhibitory at higher ones [2,154]. Hence, it is possible that, following N/OFQ administration the reduced propensity of animals to explore the mazes used for the anxiety tests is secondary to an impairment of their motor skills. If this interpretation may explain the anxiogenic effect following 1.0 nmol of N/OFQ in the EPM test, it is more difficult to understand the pro-anxiety effect of the peptide at 0.01 nmol dose in the light/dark box [122]. To interpret this latter finding, the authors suggested that N/OFQ administration could exert different effects on anxiety depending on the basal anxiety status of the animal, and how stressful the test conditions are, at the time N/OFQ is administered [122]. In line with this view, it was shown that the anxiolytic properties of N/OFQ were enhanced in rats subjected to stressful experiences. For instance, in a study in which N/OFQ was given into the CeA it exerted anxiolytic effects in restrain stressed but not in not-stressed rats [93]. 

The N/OFQ-NOP system has been also implicated in post-traumatic stress disorder (PTSD). In a translational study, PTSD symptoms in patients with childhood trauma were associated with a single nucleotide polymorphism (SNP) in the NOP receptor gene. Accordingly, mice exposed to the fear conditioning test, a model of PTSD, showed increased NOP receptor gene expression in the amygdala, whereas intra-amygdala treatment with the NOP selective agonist SR-8993 blocked the consolidation of the fear memory [155]. In line with this report, a PET study using the radioligand ^11^C-NOP-1A reported an increased NOP receptor binding in college women exposed to sexual abuse [156], suggesting a crucial contribution of the N/OFQ-NOP receptor system in modulating anxiety symptoms that follow a traumatic event. Finally, increased expression of N/OFQ was observed in the hippocampus and dentate gyrus of rats subjected to restrain and social defeat stress [99,157,158]. Altogether, these results support the involvement of the N/OFQ-NOP system in the memory and emotional aspect of traumatic events associated with PTSD. A plausible interpretation is that enhanced expression or activity of the N/OFQ-NOP system is an adaptive change aimed at contrasting the emergence of PTSD symptoms. On the other hand, the possibility that facilitation of N/OFQ-NOP transmission contributes to the emergence of PTSD signs should be also considered. For instance, in the same PET study mentioned above it was found that lower brain NOP receptor levels is associated with less severe PTSD symptoms [156]. Altogether this data indicate that N/OFQ-NOP transmission is necessary to inhibit the development of an anxious state, as corroborated by studies conducted on NOP receptor knockout animals. N/OFQ knockout (−/−) and NOP−/− mice exhibited innate levels of anxiety higher than their wild-type littermates in several tests [159,160] and NOP−/− rats displayed an anxiety-like phenotype in the EPM while showing intact mobility, indicating that ablation of N/OFQ-NOP signaling is associated with abnormal generalized anxiety but do not impair locomotor activity [161]. Altogether, the evidence discussed so far indicate that N/OFQ-NOP signaling is necessary to modulate anxiety, and that, with two exceptions [122,127], NOP agonism induces anxiolytic effects. 

The effect of NOP antagonism in modulating anxiety has also been studied.The NOP receptor antagonist UFP-101 (10 nmol, i.c.v.) counteract the anxiolytic effect of N/OFQ (0.5–10 nmol, i.c.v.) in EPM without modifying anxiety-like behavior per se [139], whereas it showed anxiolytic properties in the elevated T-maze in the 1-10 nmol (i.c.v.) range in an inverse dose/dependent manner [162]. LY2940094 reduced freezing in the fear conditioning test in mice [163], but failed to induce anxiolytic-like effects in other behavioral assays, such as rat conditioned emotional response, mouse four plate test, rat novelty-suppressed feeding, mice marble-burying [163,164]. More recently, in a model of inescapable electric foot-shock, the NOP antagonist SB-612111 (0.1, 1.0, 10.0 mg/kg, i.p.) reversed helpless-induced anxiety-like behaviors, whereas it increased anxiety levels in non-helpless mice and it was ineffective in non-stressed animals tested in the EPM [165]. Finally, acute administration of another NOP antagonist, JC-113397 (7.5, 20.0 mg/kg, i.p.) reversed anxiety induced by a predator exposure (cat) in rats [166]. Overall these data demonstrate that NOP antagonists may exert anxiolytic-like effects, however these anxiolytic properties of NOP antagonists emerge only under stressed conditions. 

Evidence in support of a role for the N/OFQ-NOP receptor system in depression has also been published. Patients diagnosed with post-partum depression, major depressive disorder (MDD) and bipolar disorder showed increased plasma levels of N/OFQ [82,167]. However, in preclinical studies neither N/OFQ [111,168,169] nor synthetic NOP receptor agonists [143,170] were found to affect depressive-like behavior. Yet, NOP−/− rats express anti-depressive-like behavior [161], suggesting that toning down N/OFQ-NOP signaling may have anti-depressant effects. Indeed, blockade of the NOP receptor showed anti-depressive effects when tested in various depression-related preclinical and clinical studies. The first evidence of an anti-depressive effect of the NOP receptor antagonism was published in 2002 [168]. In that work, both the peptidergic [Nphe1]N/OFQ(1–13)-NH2 (25–50 nmol, i.c.v.) and non-peptidergic J-113397 (20 mg/kg, i.p) NOP antagonists reduced the immobility time in the forced swimming test in mice [168]. Later, consistent results were obtained testing three different NOP receptor antagonists, SB-612111, UFP-101, and LY2940094 in forced swimming and tail suspension tests in mice [163,169,170,171,172] and rats [164]. In addition, depressive-like behaviors induced by a chronic mild stress (CMS) model of depression were mitigated by chronic administration of UFP-101 (5.0, 10, 20 nmol, i.c.v.), showing results comparable to other antidepressants, such as fluoxetine and imipramine [124,173]. The effectiveness of NOP receptor antagonists as anti-depressants was also demonstrated in other models of depression. SB-612111 (10 mg/kg, i.p.) and UFP-101 (1.0–10 nmol, i.c.v.) attenuated depressive-like symptoms in the mouse learned helplessness model [170,174,175]. Furthermore, UFP-101(10 nmol, i.c.v.) and SB-612111 (10 mg/kg, i.p.) reversed lipopolysaccharide (LPS) induced depressive-like behaviors in mice subjected to the tail suspension test [176]. Consistently, when LPS was injected into NOP knockout (NOP−/−) mice, it failed to elicit depressive effects, whereas it markedly increased the immobility time in wild-type mice [176]. Noteworthy, the anti-depressant effect of NOP receptor antagonism has been demonstrated to translate in humans diagnosed with major depressive disorder (MDD). In a double-blind, placebo-controlled trial, 8 weeks of treatment with LY2940094 (40 mg/day) significantly reduced depressive symptoms measured by the GRID-Hamilton Depression Rating Scale [164]. In summary, both preclinical and clinical results support a role for the N/OFQ-NOP receptor system in depression and indicate that treatment with NOP receptor antagonists may be an effective strategy to treat depressive and mood disorders. 

It is interesting to note that NOP receptor antagonists showed both antidepressant and anxiolytic properties, and that as anxiolytics they are effective only in tests in which anxiety is measured by the absence of a coping behavior induced by a stressor [163,165,166]. This is similar to the rodent model of depression, in which depressive-like behavior is also measured by the absence of coping induced by a stressful and/or frustrating condition (e.g., immobility in forced swimming test and tail suspension test). This suggests that the anxiolytic-like effects given by NOP antagonists could derive from their anti-depressant properties. Indeed, it was demonstrated that, similar to NOP antagonists, antidepressants reduces anxiety-like behavior in the fear conditioning test [163,177] and in the elevated T-maze, but not in the EPM [139,162,178]. Therefore, it is possible that the anxiolytic-like effects of NOP antagonists may derive from their anti-depressant properties. 

In conclusion, a dual role of the N/OFQ-NOP receptor system in the modulation of anxiety and depression has emerged from both preclinical and clinical studies. Activation of the NOP receptor produces a reduction in the anxiety response, whereas its blockade causes antidepressant-like effects. In light of the limited efficacy of the current anxiety and mood disorders treatments, the N/OFQ-NOP receptor system represents a new compelling pharmacological target for the treatment of several mood-related disorders, including anxiety, depression and post-traumatic stress disorder (PTSD).

## 4. Role of N/OFQ-NOP Receptor System in the Interaction between Stress and Drug Addiction

N/OFQ and the NOP receptor are distributed in brain areas modulating motivation and stress, making them key components of the neurocircuitries of addiction. Thanks to its wide distribution, the N/OFQ-NOP system plays a role in the primary reinforcing properties of drugs of abuse and their interaction with the stress system. PET studies revealed increased NOP expression in stress and motivation-related brain areas of cocaine addicts [179] but not of human alcoholics [180]. However, hypomethylation of the NOP receptor has been associated with increased psychosocial stress exposure and higher frequency of binge drinking episodes in adolescents [181]. Moreover, decreased expression of PPNOC and NOP receptors was reported in the CeA and hippocampus of postmortem brains of human alcoholics [182]. These data indicate a role for the N/OFQ-NOP system in the interaction between stress and addiction [181]. Here we will focus on this specific aspect of the role N/OFQ-NOP receptor system in addiction; for a more general and comprehensive review of the role N/OFQ-NOP system in addiction the reader can refer to [82,183,184]. 

Similar to drugs of abuse, stress-related neuropeptides and hormones target the mesolimbic system and, similarly to drugs of abuse, enhance dopaminergic transmission. This accounts for a role of stress in the reinforcing properties of drugs of abuse and for the development of drug addiction. Indeed, it was demonstrated that exposure to stress facilitates the initiation and escalation of drug self-administration and enhances vulnerability to addiction (for review see [185]). Therefore, the anti-stress properties of N/OFQ and the distribution of NOP receptors suggested that activation of this system by agonists would decrease drug self-administration. Consistent with this hypothesis, i.c.v. injection of N/OFQ attenuated alcohol self-administration in Marchigian Sardinian alcohol-preferring (msP) rats [186]; a line genetically selected for their high alcohol preference and with an innate elevated anxiety and high response to stress, that consume high amount of alcohol to alleviate their innate negative affective state [187,188]. In a follow-up paper, the same group confirmed that N/OFQ attenuated alcohol intake in msP rats while being ineffective in non-preferring Wistar rats. MsP rats showed increased N/OFQ and NOP receptor expression and ligand-receptor binding in the amygdala and found the site of action of N/OFQ to be the CeA [104]. Moreover, chronic intermittent ethanol consumption normalized the amygdalar over-expression of PPNOC and NOP genes in msP rats to Wistar level [189]. Confirming a pivotal role of N/OFQ in mediating the interaction between stress and alcohol in the CeA, N/OFQ was shown to revert CRF-enhanced GABA-mediated inhibitory post-synaptic potentials (IPSPs) in CeA slices of both naïve and ethanol-intoxicated rats [101]. N/OFQ also attenuated the expression of psychological and physical signs of alcohol withdrawal [190,191], a phenomenon leading to binge intoxication mediated by neurocircuitries pivoted in the CeA (for review see [192]). In line with exogenous delivery of the neuropeptide, peripheral administration of the NOP agonist MT-7716 reduced alcohol intake in msP rats over two weeks of treatment without sign of tolerance and the effect persisted for one additional week after treatment discontinuation [193]. Again, similar to N/OFQ, MT-7716 did not affect alcohol intake in non-dependent Wistars rats, but it was effective in post-dependent animals [194]. However, a different NOP agonist, SR-8993, could reduce alcohol self-administration and motivation, and yohimbine-induced reinstatement in non-dependent Wistar rats [144]. Data on alcohol self-administration were recently paralleled in cocaine. We demonstrated that the NOP agonist Ro64-6198 reduced escalated cocaine self-administration in msP but not in Wistar rats [195], while Cippitelli and colleagues demonstrated that another NOP agonist, AT-312, decreased escalated cocaine self-administration and cocaine hedonic set point in Sprague Dawley rats [196].

Stress is a major determinant of drug relapse in human addicts [192]. Stress induced relapse is modeled in preclinical studies by either electric foot-shock or yohimbine-induced reinstatement of drug-seeking [197]. Activation of the NOP receptor by i.c.v. injection of N/OFQ prevented foot-shock-induced reinstatement of ethanol but not cocaine-seeking [198], while the NOP agonist MT-7716 blocked foot-shock-induced reinstatement exclusively in post-dependent rats [194]. Activation of NOP receptors by synthetic agonists blocked yohimbine-induced reinstatement of alcohol-seeking in both outbred Wistars [144] and in msP rats [193].

This body of literature indicated that N/OFQ and NOP agonists decrease drug self-administration and seeking through stimulation of NOP transmission. However, recent unexpected results obtained with NOP−/− rats raised doubts on this interpretation. These animals, expressing a non-functioning NOP, contrary to expectations, showed a reduced self-administration and motivation for alcohol, cocaine, and heroin, suggesting that NOP receptor transmission is necessary to maintain drug self-administration [199]. Consistent with results in NOP knockout rats, the NOP antagonist LY2940094 prevented alcohol intake, motivation, and yohimbine-induced reinstatement in msP and Indiana (P) alcohol-preferring rats [120]; the site of action of NOP antagonism being the CeA and the VTA, as verified with LY2817412 [200]. LY2940094 was effective also when tested in a clinical set up, where it decreased depressive symptoms in major depressive disorder patients and in heavy alcohol drinkers [167]. This indicated that both NOP agonists and antagonists decrease drug consumption and seeking [167] and the mechanism of action of the N/OFQ-NOP is not as straightforward as initially thought. A possibility is that NOP agonists and antagonists could work through different micro-circuitries, but this is unlikely given our recent report that LY2817412 decreases alcohol intake through the CeA [200]), the same site of action of N/OFQ [104]. However, since no data are available at present on the effect of intra-VTA effect of NOP agonists on drug-intake and LY2817412 also worked through the VTA [200], this hypothesis cannot be completely excluded. An alternative hypothesis is that NOP agonists function as functional antagonists. Besides Gi/o, the binding of N/OFQ to NOP activates the β-arrestin 2 pathway promoting the internalization of the receptor [201]. Therefore, it could be hypothesized that NOP agonists may act as functional antagonists by promoting the desensitization of the receptor. Accordingly, only NOP agonists showing activity on the β-arrestin 2 pathway exert anxiolytic effects [150]. This would fit with the effect of NOP agonists on alcohol consumption in alcohol-preferring rats, who drink alcohol to alleviate their innate elevated anxiety [187,188], and would be consistent with our recent results showing that LY2817412 blocks alcohol intake in msP rats through the CeA [200]. 

## 5. Role of the N/OFQ-NOP System in the Regulation of Feeding and Food-Related Disorders 

N/OFQ and its receptor are part of a complex neural network that regulates consummatory behavior, through interaction with several other neuropeptidergic systems [202]. N/OFQ and NOP receptors are largely distributed throughout the rat brain [17], including areas associated with eating behavior and energy balance [17,18]. NOP agonism regulates the activity of neurons within these sites affecting the activity of several other neurotransmitters involved in feeding regulation. Moreover, considering the important commonalities between eating disorders and drug abuse [203,204,205] and the role of the N/OFQ-NOP system in the modulation of motivated behaviors (see paragraphs above), it is conceivable that the function of the N/OFQ-NOP system is not limited to the metabolic regulation of feeding and in the induction of endogenous antioxidants under certain conditions, such as diabetes mellitus [206]. Indeed, it plays a role in the motivation for food, possibly contributing to the etiopathology of eating disorders. 

In 1996, Pomonis et al. reported that central administration of N/OFQ promotes food intake in rodents [21], and the intra-hypothalamic arcuate nucleus (ARC) injections revealed to be the most powerful to induce hyperphagia [207]. Later, it was shown that this hyperphagic effect was long-lasting and robust in rats maintained on regular diet [208,209], or on a moderately high-fat diet (HFD) [210]. Moreover, in mice, chronic i.c.v. administrations enhanced both food intake and body weight especially in HFD animals [210]. An electrophysiological study from VMN neurons observed that N/OFQ, through the activation of inwardly rectifying potassium (GIRK) current, inhibited leptin receptor expressing VMN neurons that would result in a suppression of their anorexigenic output [211]. 

Recently, the hyperphagic effect of N/OFQ was associated to the inhibition of critical neuronal activity, such as the neurotransmission at synapses involving ARC POMC neurons and steroidogenic factor (SF)-1 neurons in the VMN, particularly in males and under HFD condition (see details in [67,212,213]. To note, this anorexigenic neurotransmission is implicated in the modulation of homeostatic energy balance because, if activated, attenuates food intake and improves energy expenditure [214,215,216,217,218]. Remarkably, long exposure to HFD increases also the excitability of ARC N/OFQ neurons [219], leading to an increased sensitivity of VMN SF-1/ARC POMC synapses to the inhibitory action of N/OFQ and thus to a significant overconsumption [213].

Earlier work has also shown that at orexigenic doses of N/OFQ significantly decreased body temperature for at least one hour after intrahypothalamic injection. This effect was attributed to the ability of the peptide to modulate the activity of warm-sensitive neurons in the preoptic area of the anterior hypothalamus [220]. Considering the concomitant stimulation of consummatory behavior and the decrease in body temperature it was suggested that N/OFQ act as an “anabolic” neuropeptide regulates energy balance [221]. 

In response to repeated food restrictions, the expression of N/OFQ and NOP receptor transcripts are significantly down-regulated in the BNST and increased in the ventromedial hypothalamus and in the VTA [222]. The orexigenic effect of N/OFQ is increased following episodes of food restrictions [222]. Interestingly, central administration of N/OFQ stimulates c-Fos expression, a marker of neuronal activation, in the PVN and in extrahypothalamic sites like the solitary tract, the CeA, the lateral septal and habenular nuclei [223]. C-Fos experiments also showed that intracranial administration of N/OFQ reduced the activation of both oxytocin neurons in the PVN [224] and α-melanocyte-stimulating hormone (α-MSH) cells in the ARC [225].

Together these results on one hand suggest that N/OFQ function is modulated by the feeding condition and on the other indicate that orexigenic effects of the peptide are probably linked to the inhibition of hypothalamic satiety signals such as those mediated by oxytocin and α-MSH systems (see review [226]). Oxytocin, a pituitary neuropeptide hormone largely expressed in the PVN, is an important anorectic mediator linked to the regulation of caloric intake, gastric emptying and, as recently discovered, involved in the modulation of binge eating [227,228]. The α-MSH is instead an anorexigenic neuropeptide, synthesized by neurons located in the lateral part of the ARC, which promotes satiety-induced termination of feeding and increases energy expenditure [229,230]. 

Additionally, N/OFQ might induce hyperphagia by inhibiting the endogenous cocaine and amphetamine-regulated transcript (CART) [231,232,233], which is another anorectic peptide also regulated by leptin [234]. Beyond the modulation of these hypothalamic mechanisms, N/OFQ modulates the activity of 5-HT neurons in the DRN (see above) which may also change feeding responses. Stimulation of 5-HT transmission decreases food intake in animals and drugs acting as agonists for this system have been proposed for the treatment of body weight gain and obesity [235,236,237,238]. 

An alternative possibility is that the orexigenic effects of N/OFQ depend on the enhanced motivation for food mediated by reward related mechanisms. This alternative hypothesis is in part supported by a recent very elegant study demonstrating that the reinforcing and rewarding properties of highly palatable food intake (HPF) are linked to the activity of N/OFQ transcript expressing cells in the CeA [239].

Recently, using a preclinical model of binge-like eating behavior in female rats [240], triggered by yo-yo dieting and stress [237] it was observed that at low doses (0.5 nmol/rat, i.c.v.) of N/OFQ selectively reduced HPF intake [222]. However, at higher dose (1 nmol/rat, i.c.v.), HPF consumption was increased, following food restrictions [222] and epigenetic modifications of N/OFQ and CRF systems were detected in binge eating rats [241]. Notably, binge eating studies in rodents were also carried out to test the effect of LY2940094 and SB 612111, two selective NOP receptor antagonists. Results showed that both molecules markedly reduced HPF consumption in rodent models [242,243], whereas basal chow food intake is not affected by NOP antagonism [242]. Consistently, as mentioned above, PNOC-expressing neurons in the ARC revealed to be activated by HFD and to inhibit the anorexigenic POMC neurons [219], leading to hyperphagia. Accordingly, N/OFQ injected into the ARC exacerbates binge eating episode on HFD [67]. On the other hand, N/OFQ administered into the VTA reduced binge eating, inhibiting the reward-encoding A_10_ dopamine neurons, in obese but not lean males, and in both lean and obese females [67]. These findings suggest that VTA-specific actions of N/OFQ are influenced by the diet and sex. Similarly to N/OFQ-NOP system, considerable evidence suggested sex differences in other orexigenic signaling mechanisms implicated in binge eating, such those depending on the relaxin-family peptide-3 [244,245] and orexin-1 receptors [246,247,248]. This is not surprising considering that sexual dimorphism has been described in binge eating behavior both in humans [249,250,251] and animals [252,253,254,255,256]. In light of the complex role of N/OFQ in feeding regulation, and based on the current knowledge, it is difficult to provide an unambiguous interpretation of the effect of NOP on binge eating. 

Nonetheless, it is worth reminding the strong association between obesity [257,258], eating disorders and depression. Hence, considering the antidepressant properties of NOP antagonists it is tempting to speculate that they may not only directly act by blocking the orexigenic effect of N/OFQ [23,169,259] but they can also indirectly attenuate eating disorders symptoms by relieving from negative affect (i.e., depressive-like symptoms) [56,124,173,260]. In this respect, it is worth mentioning that fluoxetine, an antidepressant acting as a selective serotonin reuptake inhibitor (SSRI), is efficacious in attenuating carbohydrate craving, bulimia nervosa, and pathological eating attitudes in humans and binge eating in rodents [240,261,262]. 

## 6. Conclusive Remarks

Evidence indicates that the N/OFQ-NOP receptor system plays a significant role in the modulation of stress, mood and motivation. The three primary modulatory actions of N/OFQ are: the functional antagonism towards the CRF mediated transmission at extrahypothalamic sites; the modulation of HPA axis activity and the control of the catecholaminergic transmission within the mesolimbic system. Thanks to its role as stress modulator the N/OFQ-NOP system has emerged as potential therapeutic target to treat stress-associated psychiatric disorders. For instance, clinical evidence supporting the efficacy of NOP antagonists in depression has been obtained. On the other hand, a wealth of preclinical data support the efficacy of NOP agonists in the treatment of anxiety and stress-induced relapse to drug seeking in animal models of substance use disorder. The therapeutic potential of NOP agonists in addiction is further corroborated by the fact that the N/OFQ-NOP receptor system also modulates mesolimbic dopamine transmission acting as a reward stop signal thus reducing the intake of drugs of abuse. Notably, recent data indicate that the consumption of these substances is also reduced following NOP blockade, suggesting a complex role of the N/OFQ-NOP system in drug abuse. Lastly, this peptidergic system seems to play an intricate role in feeding with the possibility that eating disorders such as bulimia nervosa and binge eating are linked to its dysregulation. In this respect preclinical data support the therapeutic potential of NOP antagonism.
